# *Staphylococcus aureus* From Prosthetic Joint Infections and Blood Cultures Display the Same Genetic Background

**DOI:** 10.1111/apm.70038

**Published:** 2025-07-03

**Authors:** Bo Söderquist, Peter Wildeman, Marc Stegger, Bianca Stenmark

**Affiliations:** ^1^ School of Medical Sciences, Faculty of Medicine and Health Örebro University Örebro Sweden; ^2^ Department of Orthopedics, Faculty of Medicine and Health Örebro University Örebro Sweden; ^3^ Department of Laboratory Medicine, Faculty of Medicine and Health Örebro University Örebro Sweden; ^4^ Department of Bioinformatics Statens Serum Institut Copenhagen Denmark; ^5^ Antimicrobial Resistance and Infectious Diseases Laboratory Harry Butler Institute, Murdoch University Perth Australia

**Keywords:** hema‐togenous infections, prosthetic joint infections, *Staphylococcus aureus*, whole‐genome sequencing

## Abstract

Hematogenous prosthetic joint infections (PJIs) are primarily associated with *Staphylococcus aureus* , and there is a 30%–40% risk of contracting a hematogenous PJI following an *S. aureus* bacteremia. The aim of this study was to investigate whether identical strains of *S. aureus* were present in each patient from a cohort with both bacteremia and PJI and to explore the genomic differences between paired isolates obtained from blood cultures and tissue biopsies. All patients with a PJI and a temporally concomitant bacteremia due to *S. aureus* from 2005 to 2020 were included. Paired isolates of *S. aureus* from tissue biopsies and blood cultures were subjected to whole‐genome sequencing. Twenty‐four episodes of PJI were identified in 23 patients. All pairwise isolates from individual patients belonged to the same multilocus sequence type, clonal complex, and core genome multilocus sequence typing (cgMLST) complex type. The median number of single nucleotide polymorphisms (SNPs) in the conserved core genomes between the pairwise isolates was 3. In conclusion, identical cgMLST complex types and low levels of SNP differences between paired isolates of *S. aureus* from blood cultures and tissue biopsies suggest hematogenous seeding in all cases of PJI in this cohort.

## 1 Introduction

Arthroplasty surgery is a common procedure that has significantly improved the quality of life for many patients. The results of primary total hip and knee replacement surgery are usually excellent, with restored joint function and pain relief. However, serious complications such as prosthetic joint infection (PJI) occur in approximately 1%–2% of cases [1, 2]. The growing proportion of older people in the world will increase the need for arthroplasty surgery [3, 4], and hence a larger number of patients will be living with a hip or knee replacement. In Sweden, 3.3% of the population already has at least one hip or knee arthroplasty [5]. As a result, more individuals are at risk of contracting a hematogenous seeding of the prosthetic device during transient or persistent bacteremia.

The majority of PJIs will present within the first 90 days following arthroplasty surgery, but patients will be at risk for the entire lifespan of the prosthetic joint [1, 2, 6]. Most of the later‐developing cases probably represent dissemination to the prosthetic joint devices via the hematogenous route.

The most common bacteria causing PJIs are staphylococci, including both *Staphylococcus aureus* and coagulase‐negative staphylococci (CoNS), predominantly *Staphylococcus epidermidis* ; together, these species account for 55%–75% of all PJIs [7, 8, 9]. Late, but acute, hematogenous PJIs are primarily associated with *S. aureus* [6, 10]. The risk of contracting a hematogenous PJI following a *S. aureus* bacteremia has been reported to be as high as 30%–40% [11, 12].

The present study compared paired samples of *S. aureus* isolated from blood cultures and deep tissue biopsies of patients with PJIs by whole‐genome sequencing and subsequent genomic analyses. The aim was to investigate whether identical strains of *S. aureus* were present in each patient, and, if so, to explore the genomic differences between the paired isolates of *S. aureus* obtained from blood cultures and tissue biopsies.

## 2 Materials and Methods

### 2.1 Bacterial Isolates

The study included all patients with PJI and temporally concomitant bacteremia due to *S. aureus* with stored isolates from tissue biopsies and blood cultures at the Department of Laboratory Medicine, Clinical Microbiology, Örebro University Hospital, Sweden, from 2005 to 2020. Patients eligible for inclusion were identified through a database search for PJI at the Department of Laboratory Medicine, Clinical Microbiology, which is the only clinical microbiological facility in the region of Örebro County. Multiple tissue biopsies obtained from arthroplasty patients were registered with a specific code in the laboratory. Patients with ≥ 2 positive tissue cultures with growth of *S. aureus* were also searched for positive results from blood cultures. The patients' medical records were examined to collect information about the cohort. Clinical parameters, demographics, and laboratory findings were recorded at the time of PJI diagnosis.

Tissue biopsies, usually five, and one synovial fluid sample were obtained intraoperatively per protocol during debridement and revision surgery or by ultrasound‐guided biopsies, and were per routine cultured on GC agar (GC Medium Base, Becton Dickinson, Sparks, Maryland, USA, supplemented with 1% BBL IsoVitaleX enrichment) and incubated in air with 5% CO_2_ at 36°C, on fastidious anaerobe agar (FAA) plates (4.6% LAB 90 FAA, LAB M, Heywood, UK) supplemented with 5% horse blood (v/v) and incubated at 36°C in an anaerobic atmosphere, and in fastidious anaerobic broth (FAB) (2.97% FAB, LAB M, supplemented with 1% D‐glucose) incubated at 36°C. Aerobic and anaerobic blood cultures were obtained using the BACTEC system (Becton Dickinson, Franklin Lakes, NJ, USA). Species identification was performed by matrix‐assisted laser desorption ionization—time of flight mass spectrometry (Microflex LT and Biotyper 3.1; Bruker Daltonics, Bremen, Germany).

Antibiotic susceptibility was determined per routine by disc diffusion, performed and interpreted according to European Committee on Antimicrobial Susceptibility Testing (EUCAST) guidelines (http://www.eucast.org).

Isolates were stored in preservation medium (trypticase soy broth with 0.3% yeast extract and 29% horse serum) at −80°C.

### 2.2 Genome Sequencing and Analyses

DNA extraction was performed using the QIAsymphony DSP Virus/Pathogen Midi kit (Qiagen GmbH, Hilden, Germany) with the QIAsymphony system (Qiagen) according to the manufacturer's instructions, with RNase treatment and elution in Tris–HCl (pH 8). The isolates were whole‐genome sequenced using the Nextera XT DNA Library Preparation Kit (Illumina) or Illumina DNA Prep (Illumina) on a MiSeq (Illumina) with a read length of 2 × 250 bp or 2 × 300 bp and a minimum average sequencing depth of 50.

### 2.3 Molecular Typing

Sequences were trimmed to an average Phred quality score of 30 in 20 bp windows, assembled using optimized k‐mer size, downsampled to 120× using version 1.1.04 of Velvet, and assigned multilocus sequence type (ST), clonal complex (CC), and core genome multilocus sequence typing (cgMLST) complex type [13] using version 7.2.0 of SeqSphere+ (Ridom GmbH, Münster, Germany). Assembly files of isolates with novel STs were submitted to pubMLST.org for assignment of new STs [14].

Resistance determinants and toxins were investigated by uploading the raw sequences to 1928 Diagnostics (version 2023–04.8, https://www.1928diagnostics.com).

### 2.4 Single Nucleotide Polymorphisms

Single nucleotide polymorphism (SNP) analyses were performed using NASP [15], which identifies SNPs by considering only genomic positions that are conserved across all isolates in the dataset and that meet predefined quality thresholds for coverage and base quality. The alignments were made using CP050691 (NCBI) as a closed reference. Maximum likelihood‐based phylogenetic trees were constructed using IQ‐TREE with ModelFinder and bootstrap analysis using 100 replicates and subsequently visualized with metadata in version 21.0.5 of CLC Genomics Workbench (Qiagen).

Gene annotation of each SNP position was determined in Artemis [16] using *S. aureus* PMB196‐1 (GenBank accession number CP050691) as the reference. Genes in which SNPs were found were functionally annotated using version 2 of eggNOG‐mapper [17] and grouped into clusters of orthologous groups (COG).

### 2.5 Statistics

In the presentation of the results, continuous variables are expressed as mean or median, and skewed patient variables including body mass index, C‐reactive protein, and leukocyte particle concentration are presented as median and range. To investigate the correlation between the number of SNPs and days between sampling of *S. aureus* obtained from blood culture and tissue biopsy, a linear regression was performed using version 9 of GraphPad Prism (GraphPad Software, Boston, MA, USA).

## 3 Results

Twenty‐three patients were identified with a PJI in the hip or knee due to *S. aureus* . One patient had two episodes of PJI affecting the right and the left hip, respectively, with an interval of 523 days between the two episodes. The demographic data of the patient cohort are presented in Table 1. The median age was 79 years (range: 39–92 years) and the American Society of Anesthesiologists physical status classification (ASA class) was ≥ 3 in 16/24 (67%) episodes. The hip joint was affected in 17/24 (71%) cases. The median interval between the sampling date for blood cultures and tissue biopsies with growth of *S. aureus* was 3 days, with a range of 0–950 days (Table 2). In four cases, the tissue and blood cultures were obtained on the same day; in the remaining patients, all blood cultures preceded PJI samples.

**TABLE 1:** Characteristics of 24 episodes of hip and knee prosthetic joint infections (PJIs) in 23 patients with paired samples of *S. aureus* isolated from deep tissue biopsies and from blood cultures.

Cohort characteristics	*n* = 24 episodes^a^
Age, years	75 (39–92)^b^
BMI, kg/m^2^	27 (18–36)^c^
Sex, female	54% (13)
Hemoglobin, g/L	131 (98–151)^c^
CRP, mg/L	238 (13–447)^c^
LPK, 10^9^/L	12 (7–20)
ASA class ≥ 3	67% (16)
Active smoking	8% (2)
COPD	13% (3)
Autoimmune disease	17% (4)
Chronic renal failure	21% (5)
Congestive heart failure	21% (5)
Diabetes	29% (7)
Immunosuppression	13% (3)
Malignancy	13% (3)
Drug abuse	0% (0)
Localization	
Hip	71% (17)
Knee	29% (7)
Type of implant fixation	
Cemented hybrid	88% (21)
Hybrid	8% (2)
Uncemented	4% (1)
Type of implant	
Primary	75% (18)
Revision	25% (6)
Time lapse between primary arthroplasty and PJI, days	725 (15–6900)^c^
Symptoms at diagnosis	
Wound secretion	33% (8)
Pain	96% (23)
Fever	75% (18)
Redness	46% (11)
Sinus tract	0% (0)
Polymicrobial infection	4% (1)

*Note:* Data are presented as percentages and numbers, unless otherwise indicated.

Abbreviations: ASA class, American Society of Anesthesiologists physical status classification; BMI, body mass index; COPD, chronic obstructive pulmonary disease; CRP, C‐reactive protein; LPK, leukocyte particle concentration.

^a^ 24 PJI episodes in 23 patients.

^b^ Data presented as mean and range.

^c^ Data presented as median and range.

**TABLE 2:** Pairwise comparison of *S. aureus* isolated from 24 positive blood cultures and tissue biopsies in 23 patients with prosthetic joint infections.

Patient ID	Sequence type	Clonal complex	cgMLST	Days^a^	Pairwise difference		
					SNPs	Resistance markers^b^	Toxins^c^
1	46	CC45	10,004	1	4	0	0
2	30	CC30	10,022	1	3	0	0
3_1	5	CC5	9977	427	12	*blaZ*	0
3_2	5	CC5	9977	950	16	*blaZ*	0
4	22	CC22	9976	3	3	0	0
5	12	Unknown	9984	1	1	0	0
6	30	CC30	9933	0	1	0	0
7	30	CC30	9997	171	0	0	0
8	5	CC5	10,001	228	0	*blaZ*, *rpoB* ^d^	0
9	15	CC15	10,002	943	4	0	0
10	4829	CC45	8893	8	5	0	0
11	30	CC30	8856	5	2	0	0
12	5	CC5	8895	20	0	0	0
13	97	CC97	8890	14	3	0	0
14	45	CC45	8831	0	1	0	0
15	34	CC30	8838	0	1	0	0
16	30	CC30	8835	2	0	0	0
17	133	Unknown	8839	7	5	0	0
18	130	Unknown	8888	0	6	0	0
19	7	Unknown	8862	2	0	0	0
20	9103	Unknown	8895	3	4	0	0
21	8989	Unknown	23,465	10	3	0	0
22	25	Unknown	23,464	2	1	0	0
23	45	CC45	23,466	1	3	0	0

Abbreviations: cgMLST, core genome multilocus sequence typing; SNPs, single nucleotide polymorphisms.

^a^ Days between blood culture and tissue biopsy.

^b^ Predicted by 1928 Diagnostics.

^c^ Toxic shock syndrome toxin‐1, Panton‐Valentine leukocidin, and exfoliative toxins.

^d^ *rpoB:*ins475H.

**FIGURE 1:**
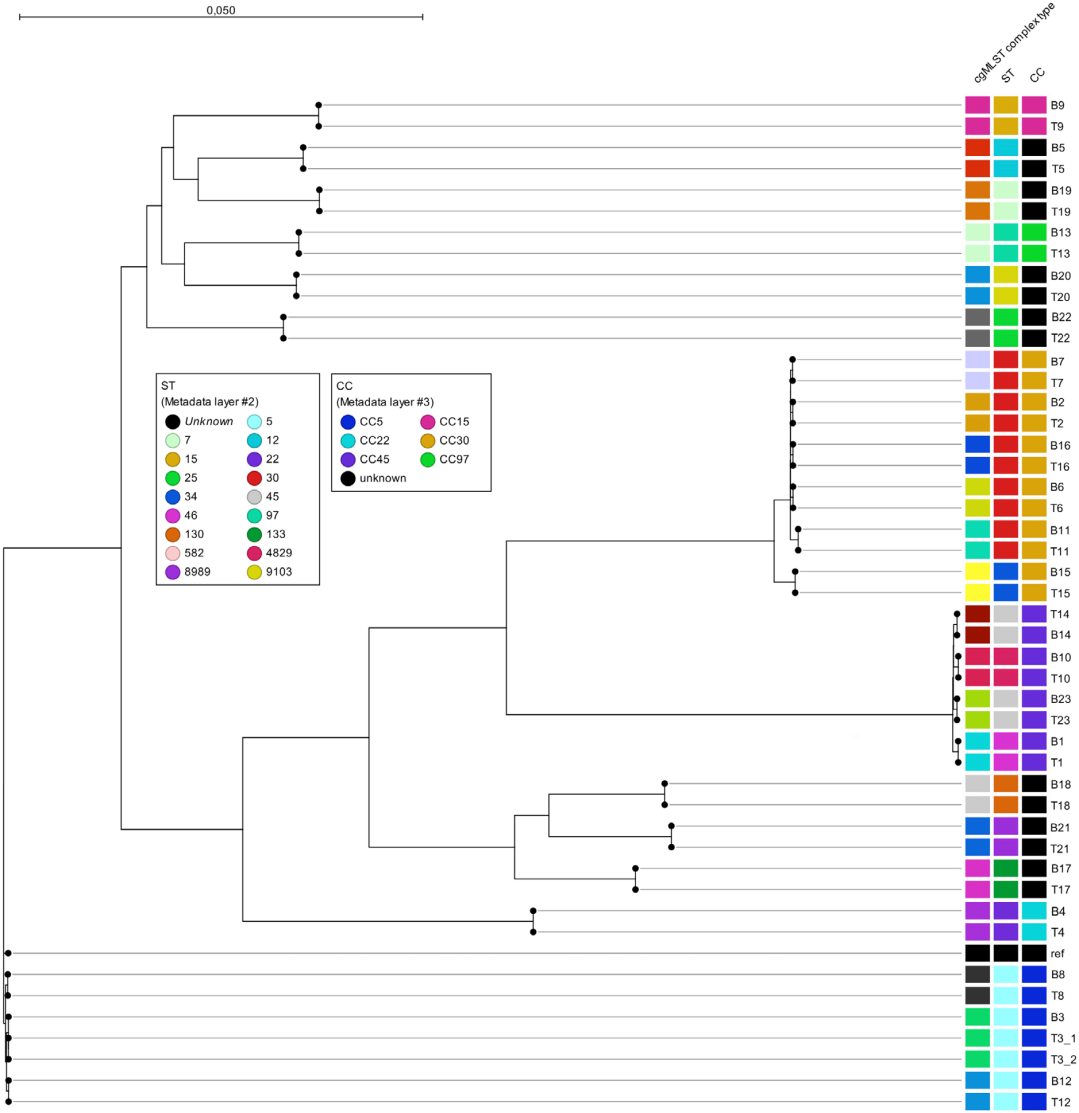
Comparison (based on 99,065 single nucleotide polymorphisms) between the 24 paired *S. aureus* samples from blood cultures (B) and tissue biopsy (T) causing bacteremia and prosthetic joint infection in 23 patients. Core genome multilocus sequence type (cgMLST) complex type, sequence type (ST), and clonal complex (CC) are shown as metadata.

All pairwise isolates from individual patients belonged to the same ST, CC, and cgMLST complex type (Figure 1 and Table 2). The most common CCs among the paired samples were CC30 (*n* = 6), CC5 (*n* = 4), and CC45 (*n* = 4). All isolates were MSSA, except for the isolates obtained from patient ID 18, which all belonged to CC130 and carried the *mecC* gene.

To further assess the genome variability between the paired isolates, SNP analysis was performed. The median number of SNPs in the conserved core genomes across all isolates between the pairwise isolates was 3, and the maximum number was 16.

A phylogenetic tree was constructed to illustrate the SNP‐based genomic similarities between the isolates (Figure 1). The genes in which the SNPs (*n* = 78) were observed are shown in Table S1. These SNPs were mainly located in noncoding areas of the genomes (Table S1) and in genes with unknown functions (Figure S1). There was no correlation between the number of days between sampling and the number of SNP differences between the blood culture and tissue biopsy isolates (Figure S2).

The presence of toxins and resistance determinants was also investigated using the assembled genomes. No isolates harbored the toxin genes *eta*, *etb*, and *lukF*‐PVL and *lukS*‐PVL [18], which encode exfoliative toxin A, exfoliative toxin B, and Pan‐ton‐Valentine leukocidin, respectively (Table S2). Ten isolates (obtained from five patients) carried *tst*, encoding toxic shock syndrome toxin‐1, but no differences were found in the pairwise comparison (Table S2).

The isolates of two patients (patient IDs: 3 and 8) differed in pairwise comparison regarding the absence or presence of the plasmid‐encoded *blaZ* gene, which encodes β‐lactamase. Otherwise, *blaZ* was consistently present in the pairwise isolates from 17/21 patients. In addition, multiple *S. aureus* isolates from patient ID 3 displayed not only diverse prevalence of *blaZ* but also varying susceptibility to rifampicin and ciprofloxacin, as reported by phenotypic antibiotic susceptibility testing. However, this patient (who experienced two episodes of PJI) showed no more than 16 SNP differences per episode in the pairwise comparison, and only 18 in total (Table S1).

Patient ID 8 had an insertion in the *rpoB* gene (*rpoB*:ins475H) encoding rifampin resistance in the tissue isolate, but not in the blood isolate. There were 228 days between the two samples, and the patient had been treated with rifampicin and ciprofloxacin due to suspected PJI without diagnostic procedures until a late debridement was performed. The same discrepancy was observed in the phenotypic antimicrobial susceptibility test.

## 4 Discussion

The present study showed identical STs, CCs, and cgMLST complex types in all paired samples of *S. aureus* isolated from blood cultures and deep tissue biopsies from 24 PJI episodes in 23 patients. The most common CCs among these patients were CC30 and CC45, which have been previously reported as being the most prevalent among PJI patients from another center in Sweden [18] and among *S. aureus* bacteremia patients in general in our region [19]. In most cases, the interval between the sampling of blood cultures and tissue biopsies was short; therefore, it was expected that the same lineage would be present. However, in some cases, there was a time period of more than 100 days between the current bacteremic episode and subsequent PJI, yet identical lineages were found.

The presence of positive blood cultures in PJI has been explored in two previous studies [20, 21]. In one of these studies, a positive blood culture at the time of diagnosis was found in 32% of the patients with acute hematogenous infections [21]. In all cases, the results of the blood cultures matched the findings of the intraoperative cultures, of which 37.5% were *S. aureus* . The other study [22] found that 25% of the PJI patients who underwent blood culture sampling were culture‐positive, and in half of these patients, the cultures yielded *S. aureus* . The same species of microorganism was identified in 86% of the cases when blood cultures and fluid and/or tissue samples were obtained at the same occasion and in 81.5% if blood cultures were taken before surgery. In addition, patients with negative blood cultures had significantly better outcomes than those with bacteremia. Other studies have shown that patients with acute hematogenous infections due to *S. aureus* have inferior outcomes compared to patients with a nonstaphylococcal etiology [10, 21, 22].

The patients in the present study had a mean age of 75 years, compared to 69 years for primary knee and hip arthroplasties in Sweden [5]. Moreover, 67% had an ASA class ≥ 3, compared to ~20% for the primary arthroplasties [5]. This is also in accordance with a previous study [20] indicating that patients with PJI and positive blood cultures are older and have more underlying diseases, which may explain the lower rate of treatment success compared to those with negative blood cultures.

However, the nature of a late acute PJI is not always clear. During persistent or transient bacteremia, seeding of bacteria on the surface of a prosthetic device can occur, which represents the hematogenous route of transmission. Initial attachment is promoted by several bacterial adhesins that interact with extracellular matrix proteins, and an infection can then be established, followed by proliferation and production of biofilm [23]. There may also exist insidious unrecognized chronic PJIs, both postinterventional and hematogenous, which later flare up to produce a secondary bacteremia and subsequent awareness, finally leading to a diagnosis of PJI [24]. In our study, positive blood cultures preceded the diagnosis of PJI in all cases and hence also preceded the finding of *S. aureus* in tissue samples, but in many cases the time interval between the samplings was short. However, in some cases the interval was > 100 days, and it is not clear whether the previous bacteremic episode represented the event when the prosthetic device became colonized. Persistent nasal carriage of *S. aureus* , which occurs in approximately one‐third of the population [25], could present a risk when combined with an arthroplasty that may be exposed to repeated transient bacteremic episodes, or indirectly by uncomplicated overlooked infections such as superficial skin infections. In addition, some long‐term nasal carriers of *S. aureus* have been found to carry the same isolate for decades, with only minor genomic alterations [26].

Genomic analyses revealed a few SNP differences (median 3) between the paired isolates. These SNPs were mainly located in noncoding genes, genes with unknown functions, or genes involved in antibiotic resistance after antibiotic treatment. Although the number of SNPs could be expected to increase with time, no linear association could be found in this study, possibly due to the limited sample size. Interestingly, no SNPs were found in the paired isolates of two patients with 171 and 228 days between blood culture sampling and the later PJI episode. However, the SNP analysis was based on the conserved core genomes, and indeed, differences in *blaZ* and *rpoB* (belonging to the accessory genome) were found in the pairwise comparison of isolates from one of these two patients who had undergone rifampicin and ciprofloxacin treatment. In addition, in vitro loss of the plasmid‐borne *blaZ* gene during subculturing has been previously reported [27].

Another result of interest was observed in patient ID 3, who experienced two episodes of hip PJIs affecting the left and the right side, respectively, but with a time span of approximately 1.5 years between the episodes. The first episode of PJI occurred more than a year after the bacteremic event, with the growth of *S. aureus* in 4 out of 4 blood culture bottles. In comparison to the blood culture, 12 SNP differences were found in the tissue culture of the left‐hip PJI episode (1.2 years after blood culture) and 16 were found in the tissue culture of the right‐hip PJI episode (2.6 years after blood culture). The isolates from this patient showed no more than 18 in total SNP differences (Table S1); therefore, all isolates were regarded as closely related, if not identical. These findings are in line with those of previous studies on the within‐host evolution of *S. aureus*, reporting an estimated rate of 2–10 SNPs in a single genome per year [28], indicating that the isolates were indeed from the same lineage.

The present study has several limitations. The number of patients included was small, and only a few paired isolates were separated by a longer time interval. In addition, the study design was retrospective. It is also possible that other genomic alterations, such as mobile genetic elements, rearrangements, or sequencing artifacts, would have been revealed in the genomic pairwise comparison using long‐read sequencing.

## 5 Conclusions

The identical cgMLST complex types and low levels of SNP differences between the paired isolates of *S. aureus* from blood cultures and tissue biopsies suggested hematogenous seeding in all cases of PJI in the current patient cohort.

## Ethics Statement

The study was approved by the Regional Ethical Review Board of Uppsala, Sweden (ref: 2016/151).

## Consent

The bacterial isolates originated from blood and tissue cultures collected according to clinical routine. These isolates were subcultured, and no human biological material was stored. Pure clinical isolates were preserved as per clinical routine.

## Conflicts of Interest

Bo.S. has been a member of an advisory board at ADVANZ PHARMA, and has also received speaker's fees from Correvio Pharma Corp and ADVANZ PHARMA. All other authors: none to declare.

## Supporting information


Figure S1.

Figure S2.

Table S1.

Table S2.

## Data Availability

All genomic data of the Swedish isolates used for this study have been deposited at the European Nucleotide Archive (https://www.ebi.ac.uk/ena/browser/home) with study accession number PRJEB74467. The dataset with all identifying information removed is available from the corresponding author upon reasonable request.
