# Supplementary figures and images for: Staphylococcus aureus From Prosthetic Joint Infections and Blood Cultures Display the Same Genetic Background
Source: APMIS. 2025 Jul 3;133(7):e70038. doi: 10.1111/apm.70038 (PMC12232108; doi:10.1111/apm.70038)

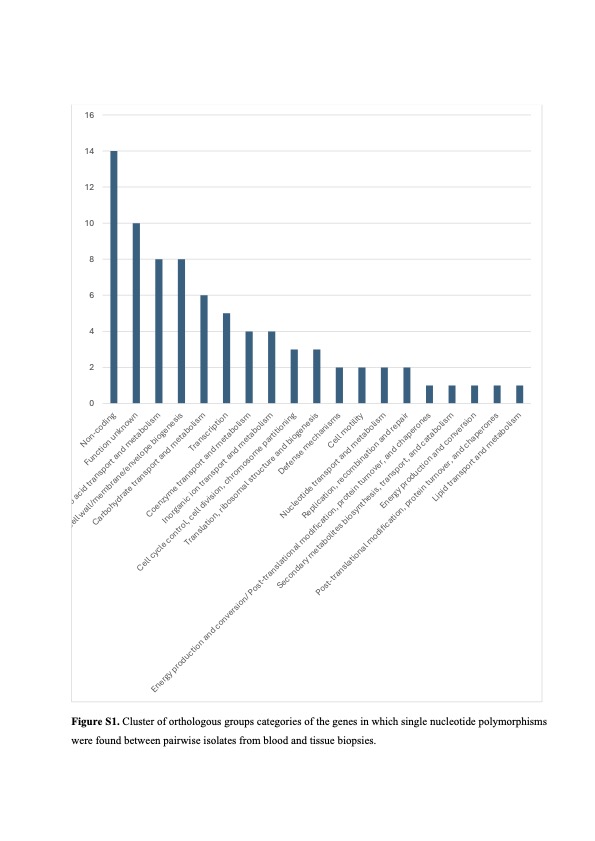

Supplement: Supplementary file 1 — Figure S1. [file APM-133-0-s001.jpg]

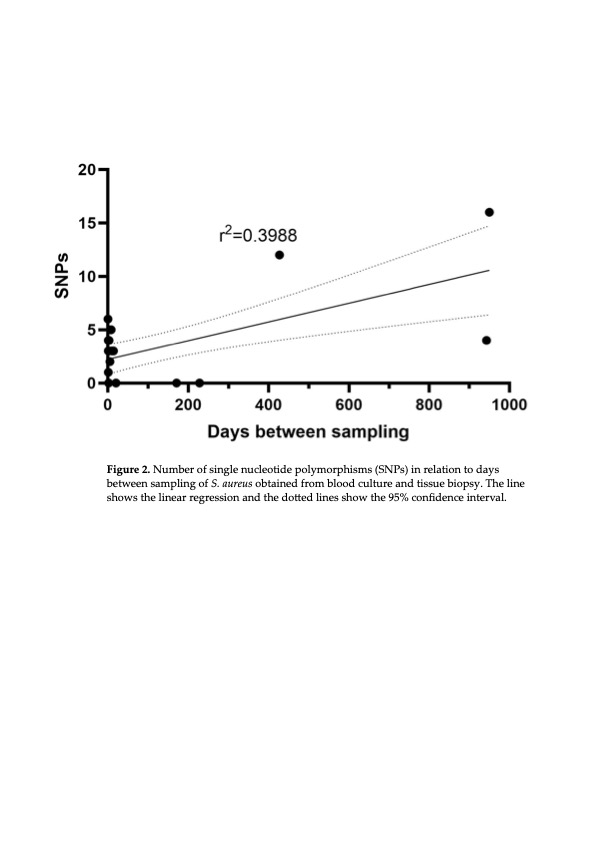

Supplement: Supplementary file 2 — Figure S2. [file APM-133-0-s003.jpg]
